# Analysis and research on the influence of music on students’ mental health under the background of deep learning

**DOI:** 10.3389/fpsyg.2022.998451

**Published:** 2022-10-12

**Authors:** Tianying Wang, Ying Zhao, Maoyuan Yin

**Affiliations:** School of Music and Dance, Mudanjiang Normal University, Mudanjiang, China

**Keywords:** analysis of music, mental health, college students’ influence, deep learning, music psychology

## Abstract

This paper makes a detailed analysis and discussion on the impact of music appreciation on college students’ mental health and the influence of music appreciation on students’ mental health, mental energy and mental structure. There has long been the idea of music promoting people’s mental health, as well as related research in the field of music psychology. For this specific group of primary and secondary school students, it should be said that it is relatively rare to consider using music education to promote their mental health. This paper summarizes the advantages of deep learning over shallow learning, explains the necessity of introducing deep learning, and describes the data representation of deep learning and several typical deep learning models. This study adopts the method of multi-evidence to conduct in-depth research and analysis. On the basis of in-depth study and research, this paper analyzes and studies the impact of music on students’ mental health, so as to lay a foundation for future research on students’ mental health. In terms of influencing factors and strategies to promote students’ in-depth learning, we should apply the research results to specific teaching situations with the help of advanced digital technology, and strive to combine theory with practice. The research shows that college students’ mental health is an important part of quality education in Colleges and universities, and music education plays an important role in the implementation of quality education.

## Introduction

With the gradual advancement of quality education concept in schools, more and more attention has been paid to the influence of music on students’ mental health in teaching. Music becomes a channel for students to go to mental health, which allows them to cultivate students’ aesthetic sentiment, enlighten their wisdom, enhance their psychological quality, enhance their aesthetic taste and construct their own spiritual framework during their growth. With the deepening of research, deep learning has not only increased a lot of related research, such as neural network, learning process, classroom teaching, etc., but also the interdisciplinary research on deep learning has developed rapidly, such as pedagogy, physics, mathematics and other disciplines. Under the background of core literacy-oriented curriculum reform, a large number of primary and secondary schools are putting in-depth learning into specific curriculum teaching practice. With the popularization of higher education, music, as an important aspect of quality education, gets more and more attention. Many colleges and universities include music appreciation in humanistic quality education courses, and put forward higher requirements for music appreciation courses. Music, as a kind of culture, has a long history in the longitudinal direction, and is vast in the horizontal direction. It plays an extremely important role in the quality of talents (Awais et al., 2020). Mental health, as an important index reflecting personal health, has been paid more and more attention in social functional departments, such as education, life and production, and has been vigorously publicized and popularized as an important part of humanistic care. The mental health of college students is one of the focuses of the current society. It is a way worth exploring to use music to adjust the mental state of college students. The mental health of college students is one of the focuses of the current society. Using music to adjust the mental state of college students is a way worth exploring (Nachmani et al., 2018). With the deepening of research, deep learning has attracted the attention of researchers in the field of education. Educational researchers have found that “learning can also be divided into depth and depth” and that deep learning is an effective way to deeply process knowledge and information and improve learning efficiency. Therefore, developing deep learning has become an important measure of contemporary learning science. At the same time, society requires citizens to master accurate information and have the ability to deal with it, so as to serve the contemporary society. Deep learning means that on the basis of understanding, learners learn new ideas and knowledge critically, integrate them with the original cognitive structure, connect many ideas with each other, and transfer existing knowledge to new situations, so as to make decisions and solve problems. At present, digital, networked and mobile new learning methods have emerged one after another and become popular. Even because of the misuse of new learning tools and technologies, some learning activities only stay at the shallow learning level, so the network is even considered as a hotbed for shallow learning, which is not suitable for deep learning activities. At present, the rapid development of economy and the fierce competition in the information society also bring great social pressure to their lives. For the current college students, they cannot bear such great pressure. This invisible pressure makes them prone to excessive self-esteem, stronger self-inferiority, easy self-centeredness, emotional, weak sense of responsibility and other psychological problems, which lead them to commit crimes easily (Kaluarachchi et al., 2021).

The early study of in-depth learning showed concern for the learning process, learning methods and learning results. At the same time, from the perspective of psychological research, the concept of learning quality with “understanding” as the core was put forward, and it was tried to describe it in a measurable way. Under the background at that time, the research trend of learning process, learning methods, learning results and quality and teaching effectiveness was a positive and concrete attempt to respond to the educational problems in the social change from the micro-level of classroom teaching, taking the educational process as the research foothold. In terms of computational complexity of network structure, when a network structure with depth k can express a certain function compactly, when a network structure with depth less than k is used to express the function, it may be necessary to increase the number of computational factors of exponential scale, which greatly increases the computational complexity. Generally speaking, for a given number of training samples, if there is a lack of other prior knowledge, people prefer to use a small number of computing units to establish the “tight expression” of the objective function to obtain better generalization ability. When the network depth is not enough, this tight expression may not be established at all. Because theoretical research shows that the function that can be compactly expressed by the network with depth *K* sometimes requires exponential growth of computing units when expressed by the network with depth *k* – 1.

In this paper, the corresponding research methods are established to analyze and explain it. In the research of deep learning, the corresponding model diagram and algorithm formula are established. In the research of music’s influence on students’ mental health, data graph and other methods are established to analyze it.

The main contribution of this paper is to conduct in-depth research and analysis of its research by using multi-evidence method. And use the method of demonstration to study and explain its research. On the basis of in-depth study, this paper analyzes and studies the impact of music on students’ mental health. Lay a foundation for the future study of students’ mental health. This paper summarizes the advantages of deep learning over shallow learning, explains the necessity of introducing deep learning, describes the data representation of deep learning and several typical deep learning models, such as convolutional neural network, DBN, and stack self-coding network, explains the reasons that may lead to the difficulties of deep learning training, introduces effective training methods, and from the aspects of initialization method, the selection of network layer and activation function, model structure This paper summarizes the new progress of deep learning research in recent years from four aspects: learning algorithm and practical application.

This paper is divided into five sections. The first section of this paper expounds the research background of the influence of music teaching on students’ mental health. The second section makes an empirical analysis of how to use network technology to support and promote deep learning. The third section studies the basic connotation of deep learning. The content of deep learning algorithm is described. Section “results and analysis” studies the influence of music on students’ mental health, and describes the research on the influence of music appreciation on students’ mental health. Section “summary” summarizes the full text. This paper summarizes the advantages of deep learning over shallow learning, and explains the necessity of introducing deep learning.

## Related work

At present, the importance of education informatization is insufficient, and there is no in-depth understanding of the positive role of university informatization construction on the development of higher education. The traditional management concept and mode of thinking have seriously restricted the construction and development of university informatization. Theoretical research results are divorced from reality. This is mainly because most of the technical personnel engaged in design are not the front-line personnel of education, which leads to the disconnection between the designed works and education. The speed of information technology updating is fast. To give full play to the benefits, we must have high-quality information management personnel. Education informatization requires technical personnel who can be responsible for the design and maintenance of information systems, as well as the integration and development of various management software. Although there are many talents in this field in ordinary colleges and universities of science and technology, most of them are in teaching posts and do not engage in business management. How to avoid the complementary connection of information systems and avoid the phenomenon of “information fortress.” How to correctly and reasonably apply information technology to the process of education informatization has not been well solved. In view of many problems in the current process of educational informatization, learning research groups have conducted descriptive empirical research on how to use network technology to support and promote deep learning. However, with the increasing attention paid to this problem, with various new learning methods constantly emerging, faced with worries and doubts about superficial and impetuous learning caused by fast-food, fragmentation and miniaturization of learning, It is necessary to deeply analyze and understand the essential connotation of deep learning, and further explore the theoretical basis of deep learning from the perspective of learning science and learning psychology, which has practical theoretical significance for understanding and understanding deep learning, revealing its mechanism and forming promotion strategies. Deep learning is more expressive than shallow learning, and the increase of depth makes the local optimal solution of non-convex objective function the main factor causing learning difficulties. From the perspective of the relevance and complexity of research topics, there are differences in the starting time of deep learning research, as well as the disciplines involved in early research (educational psychology and higher education were the earliest in countries, and educational technology was the first to introduce deep learning research in China), and the relevance and complexity of research topics are quite different. From any angle, we can see that deep learning puts forward new requirements for students’ learning. It emphasizes that learning is a kind of learning different from the past. It is no longer aimed at exams, nor is it limited to simple and mechanical copying of knowledge. Instead, it requires learners to grasp, apply, synthesize, analyze and evaluate, and be able to solve practical problems in life situations and form higher-order thinking goals.

In the research, Kresovich et al. think that the potential psychological adjustment of music is very crucial, which not only edifies students’ sentiment, but also promotes the emotional communication among students, makes the interpersonal relationship among students more harmonious, cultivates the ability of mutual assistance and assistance, and promotes the healthy development of students’ mind and body (Kresovich et al., 2021). Hense et al. think that music education is not only a means of art teaching, but also plays a significant role in psychological adjustment and treatment. Therefore, music education should play an active role and value in disease treatment in college music education. Colleges and universities should proceed from the reality of students’ mental health, endow colorful music teaching activities with the function of psychological adjustment, and widely apply them to the practice of college students’ mental health work (Hense et al., 2018). Kegelaers et al. think that music itself has a power that other arts cannot match and surpass, and this power contains very powerful emotions. This is also a very clear affirmation of the function of music in numerous literatures (Kegelaers et al., 2021). Wang et al. thinks that the “anxiety” caused by the pressure of study, employment and competition has become a major psychological problem that plagues higher vocational students (Wang and Agius, 2018). According to Terry et al. the psychological adjustment function of music for students cannot be realized only by passive appreciation. On the contrary, passive music appreciation must be expanded into active music rhythm and interactive and cooperative music-themed activities, so that students can rediscover their own values and abilities, reconfirm themselves and accept themselves in the process of listening, discussing and expressing music, so that they can play music effectively (Terry et al., 2020). Sharma et al. thinks that the stimulation provided by music satisfies the “id”. The “ID” is the primitive instinct, the most inaccessible part of the personality, and the powerful one, which includes the survival drive and sexual drive of human instinct. When the ID is not satisfied, the individual will breed anxiety and produce a tense state. The satisfaction that the ID needs can eliminate the tension and make the individual feel happy (Sharma et al., 2020). Wang et al. think that learning is the process of learners’ complex information processing activities and cognitive construction. In essence, deep learning is a process of constructing the meaning of structural and non-structural knowledge, and it is also a complex information processing process. It is necessary to effectively and finely process the activated prior knowledge and the acquired new knowledge, that is, from awareness and analysis to synthesis, application and assimilation (Wang et al., 2020). The purpose of in-depth learning is to develop higher-order thinking ability and realize meaningful learning. Its core idea embodies important concepts in cognitive science such as understanding, construction, transfer, problem solving and reflection. Ballenberger et al. think that deep learning is no longer just the similarities and differences of learning methods and strategies, but that there are significant differences in the understanding and criticism of meaning, the connection and construction of knowledge, and the migration and application of learning. The understanding of this difference has also prompted researchers to explore the essential characteristics of deep learning from the perspectives of memory mode, knowledge system, focus and learning motivation (Ballenberger et al., 2018).

## Deep learning research

### Research on the basic connotation of deep learning

**Figure 1:**
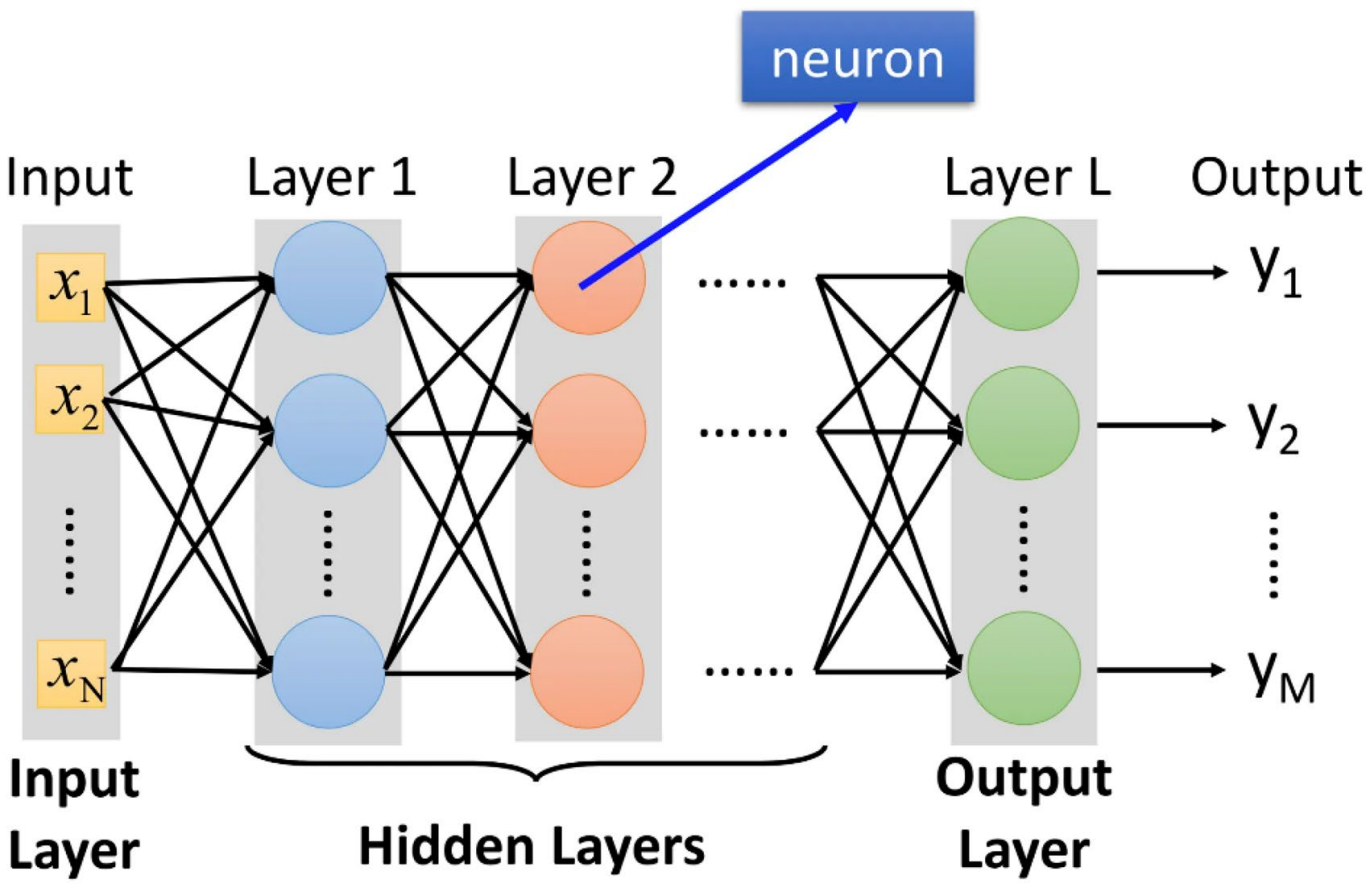
Model diagram of deep learning system.

**Figure 2:**
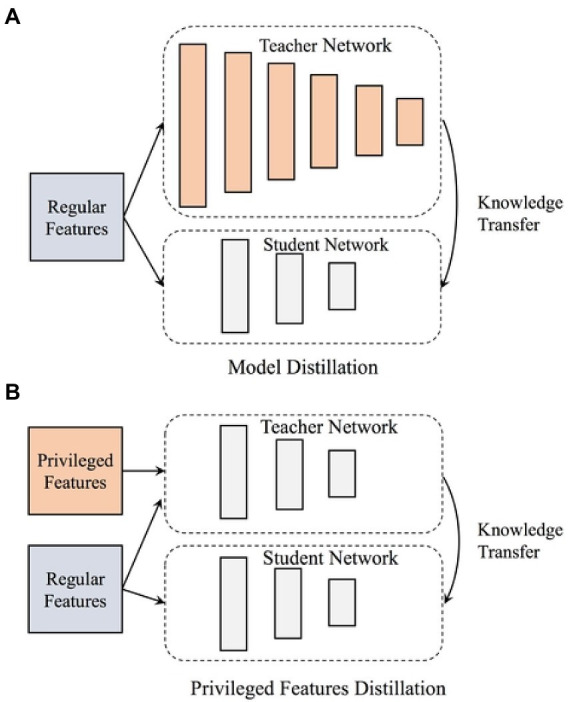
Deep learning operation model diagram. **(A)** Model distillation and **(B)** privileged features distillation.

In the 1990s, the deep learning research continued from the earlier research, which was reflected in the concern for learning results and learning process. The research at this stage mainly focuses on the topics of education, academic performance, learning strategies, learning perception and learning outcomes from the perspective of self-construction of psychological learning, while the research topics such as students, knowledge, motivation, science, classroom, mode and differences in learning quality also frequently appear. In the process of problem-solving learning, learners still pay attention to the explanation of functional level, but it includes a wider range of explanatory information, as well as more structured information and internal mechanisms. They try to understand the causal relationship between phenomena through the qualitative relationship between parts and recall association. Deep learning method tries to find the internal structure of data and the real relationship between variables. A large number of studies have shown that the way of data representation has a great influence on the success of training and learning. Good representation can eliminate the influence of changes in input data that have nothing to do with learning tasks on learning performance, while retaining useful information for learning tasks (Ascenso et al., 2018; Jensen and Bonde, 2018). Among the views on self-construction and social construction of learning, it is worth paying attention to the debate between the two research camps of symbolic processing and situational cognition for nearly 20 years. The cognitive theory of symbol processing is an important research of modern cognitive psychology. The cognitive theory of orientation symbol processing emphasizes the decisive role of knowledge on behavior and cognitive activities. It emphasizes the holistic study of cognitive processes. And “mental activity like computer” is taken as its metaphorical basis. With the method of computer simulation, a large number of simulation studies have been carried out on cognitive problems such as perceptual attention, memory and problem solving. Important progress has been made in revealing the nature and mechanism of human cognition. However, due to the limitations of metaphor itself, there are serious deficiencies in this study. The former pays attention to the processing structure of brain and thinking symbol representation, emphasizes the understanding of people’s inner mental process and the transformation characteristics of individual input and output, and pays no attention to the external environment; The latter pays attention to the structure of the external world and how it constrains and guides human behavior, emphasizing the role of history, social interaction, culture and environment, while weakening the importance of internal cognition. In order to show the overall research situation of deep learning in recent years more clearly and grasp the development trend of deep learning research, this research is based on the full-text database of Chinese academic journals in CNKI database, and adopts the method of literature analysis. Deep learning is a learning process characterized by the mental state of advanced thinking. Therefore, this paper focuses on the deep learning methods, deep learning motivation and deep learning strategies adopted by learners in the learning process. Through the statistical analysis of the frequency of high-frequency keywords, the co-cited matrix is generated, and through the advanced statistical processing such as cluster analysis, multi-dimensional scale analysis, factor analysis and social network analysis, different forms of visual graphics are drawn. According to its research, the corresponding model diagrams are established for analysis, as shown in Figures 1, 2.

**Figure 3:**
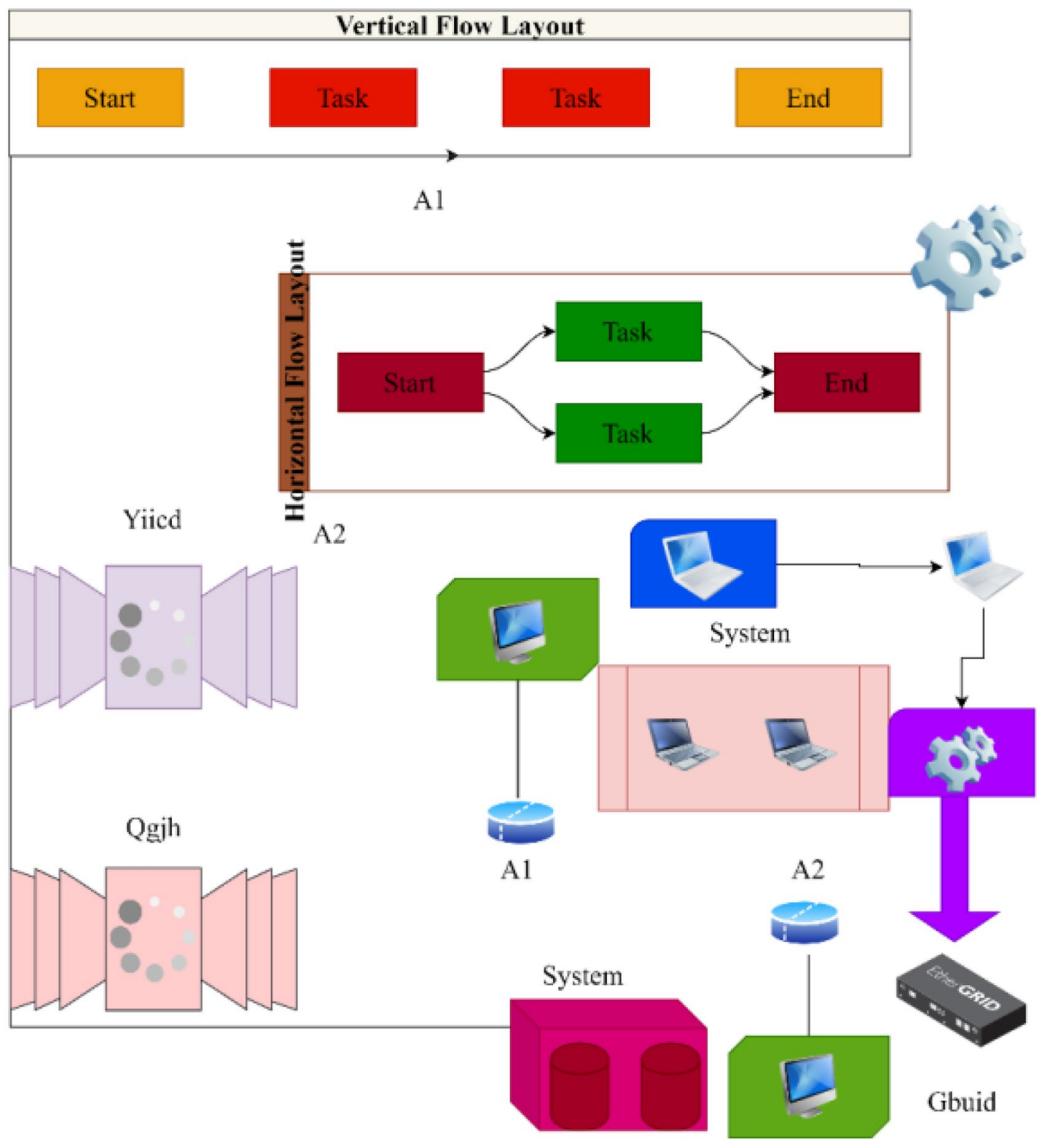
Model diagram of deep learning level.

In-depth study has stepped into the fast lane, the research results have gradually increased, and the research contents have become richer and richer. Build a virtual learning community in cyberspace, use instructional video resources or other software tools for visual learning, or conduct simulation experiments in virtual situations. Online collaborative learning, blended learning, mobile learning, and ubiquitous learning have become possible. Information-based teaching is a bilateral educational activity that educators use the current educational media, information resources and educational technology methods. The whole teaching process adheres to the teaching concept of taking learners as the main body and taking ability as the standard, “learning by doing and teaching by doing”. Information-based teaching design refers to, in order to achieve certain teaching goals, according to the characteristics of students, the theme of course content and environmental conditions. Make full use of modern information technology and resources, take learning as the center, and scientifically arrange each link and element in the teaching process, so as to realize the optimization of the teaching process. Based on the theoretical analysis and practical consensus of deep learning, it can be seen that deep learning is to solve the problems related to learning in the increasingly complex environment from the perspective of integration in explaining the rationality and experience of learning essence. Deep learning emphasizes the essence of learning. When deep learning becomes the consensus and normal state of educational practice and the essence of learning returns, the name of “deep learning” may return to “learning” instead of emphasizing “depth”. It is worth mentioning that deep learning is relative to false learning and mechanical learning, and the latter two are not what school teaching should be (Sakalle et al., 2021; Sundaresan et al., 2021). Each computing layer of the network is composed of multiple feature maps, and each feature map exists in the form of a two-dimensional plane. The neurons in the plane share the same weight set under constraints. Deep learning is a new multi-layer neural network learning algorithm. This paper analyzes the advantages of this algorithm, and on the basis of summarizing the current research situation, puts forward the existing problems in the current research. On the basis of the analysis model of deep learning constructed by the latter, the research status is summarized. As the process of deep learning mainly includes situation creation, knowledge construction, problem solving and reflective evaluation, correspondingly, these four cognitive theories also explain deep learning from these four angles. Although each has its own emphasis, it is not absolutely independent. At the same time, there are some connections among these four cognitive theories. Constructivism theory comprehensively expounds deep learning from the perspectives of learning process, results, conditions, etc. Situational cognitive theory, distributed cognitive theory and metacognitive theory also enrich and develop the related research of constructivism from many aspects. The idea of metacognition runs through all the processes of deep learning, and is involved in constructivism theory, situational cognition theory and metacognition theory. In the research, a corresponding model diagram is established to analyze it, as shown in Figure 3.

Human’s learning activity is an extremely complex system, and the study of human learning phenomenon and its essential law has always been the focus of human attention for a long time. Looking back at the long course of study and research, human beings’ exploration of learning phenomena has experienced three changes: the tradition of philosophical research, the tradition of scientific psychology based on laboratory and the tradition of multidisciplinary integration research that focuses on natural situations. A systematic and scientific learning theory has gradually formed, and gradually turned to the study and scientific research that pays more attention to complex learning phenomena in real situations. In the research of deep learning, the corresponding data tables are established for analysis, such as Tables 1, 2.

**Table 1:** Data sheet of deep learning research.

Type	Quantity	Percentage
Education	45	2.33%
Dianke	56	3.43%
Modern times	5	4.13%
Whole world	67	2.43%

**Table 2:** Deep learning specific analysis data table.

Type	Quantity	Percentage
Apply	23	53.21%
Be relevant	6	45.12%
Resources	4	45.43%

The deep learning method research includes four aspects: strategy research, teaching mode, environment design and model design. Among them, strategy research refers to various strategies and methods to promote deep learning, including information technology support strategy, learning evaluation strategy and blank space strategy. In order to meet the needs of their own development, deep learners will actively learn knowledge and skills. However, shallow learners only accept information passively in order to complete the task.

### Research on deep learning algorithm

In-depth study is interwoven with people’s way of understanding things based on rationality and experience. In the debate between self-construction and social construction of learning, cognitive objectivism moves toward the opposite side, and the irrational, socialized and contextualized parts of learning activities are gradually discovered. It can be seen from the main co-cited researchers in deep learning research that during this period, the key researchers not only include the teaching field, but also the research in multimedia learning, Computer Supported Cooperative Learning (CSCL) and other fields have received sudden attention. While discussing the advantages of information technology in promoting deep learning, people also found some problems such as learners’ cognitive load in this process. Deep learning has become a hot field in modern education. In the early stage of its development, it did not pay enough attention to it, and there is still a certain gap between the interpretation and practical application of deep learning in countries. The integration of information technology and deep learning is not high. Scholars pay more and more attention to the integration of information technology and deep learning. Improving classroom efficiency and realizing deep learning through information technology has become a hot topic (Babini et al., 2020; Cheah et al., 2020). Content analysis refers to a scientific research method that objectively, systematically and quantitatively describes the research content of a certain field, so as to deeply grasp the research status and content of the research field. Cite Space is an application software developed based on Java platform. Because it is suitable for multivariate, time-sharing and dynamic complex network analysis, it has become the most distinctive and influential information visualization software in the field of information analysis. Scientific and reasonable teaching strategies are the foundation and guarantee to make deep learning a reality. Deep learning teaching strategy is a suggestion to adjust teachers’ ideas and teaching behaviors based on the problems existing in deep learning. The research scope of deep learning mainly focuses on formal learning field and well-structured problem field. The formal field of study mainly refers to the classroom. However, learning also happens in most areas of informal learning. Informal learning, as an extension of formal learning, plays an important role in understanding and supplementing the knowledge learned in class. In the deep learning model, the convolutional neural network limits the network structure by using the local connection of the receiving domain. Another feature of convolutional neural networks is the sharing of weights. There are a lot of connected weights in the graph, but because the neurons in the same hidden layer share the same weight set, the number of free parameters is greatly reduced. The feature detection layer of convolutional neural network learns through training data, avoiding explicit feature extraction, but implicitly learning features from training data. Moreover, the neurons on the same feature mapping surface have the same weights, and the network can learn in parallel, which is also an advantage of convolutional neural network over other neural networks. The network structure of convolutional neural network is closer to the actual biological neural network, and it has unique advantages in speech recognition and image processing, especially in the field of visual image processing, and good results have been obtained. In the research, the corresponding algorithm formulas are established for analysis, such as formulas (1)–(4) plus (5), (6).


(1) P(x)=∑nlg1h1=Σnc−12sh1

(2) $$p\left[x\right]=\frac{\epsilon -2\mathrm{vC}}{2}∕\left(\bar{i}\mathrm{xcsc}\right)$$

(3) −lneiT1·rCy(x)=CyΣh3−sec1λ

(4) 12Σy¯·2=fvΩljIm∂θ1−l1

(5) $$x_{\partial \theta -1}^{\frac{{\mathrm{xl}}_{\mathrm{tnr}\gamma }^{-}}{}y_{\left(\gamma \right)}}\sum {\dot{\bar{x}}}_{1}{}^{1}\frac{3Lxi}{j^{0}}$$

(6) $$-E\frac{-1^{\prime }}{\dot{p}}⊢\partial \frac{1}{q},+\mathrm{Ep}$$

The method of random initialization is adopted for the deep neural network. The gradient-based optimization makes the training result fall into the local extreme value, but the global optimal value cannot be found. The numerical optimal solution (minimum value) trained by gradient descent method is almost equal to the analytical solution. When the parameter x to be optimized is initialized to different values, the final corresponding optimal solution (minimum value) is also different. This shows that the position of the optimal solution (local minimum) obtained by the iteration of the gradient descent algorithm is closely related to the initial value of the parameters to be optimized. With the deepening of the network structure, it is more difficult to get good generalization performance, which makes the learning result of the deep neural network after random initialization even worse than that of the shallow structure neural network with only one or two hidden layers. For deep learning, unsupervised learning and semi-supervised learning are the key components of a successful learning algorithm. Unsupervised learning makes the parameters of supervised learning enter a suitable preset area, and a good solution can be obtained by gradient descent in this area. Unsupervised learning is used in each layer of deep structured neural network to decompose a problem into several sub-problems related to the extraction of multiple representation levels, and visual learning support is provided at appropriate stages. From the perspective of visualization, the teaching process has gone through two stages, namely, the process of concretizing abstract knowledge and the process of expressing concrete knowledge in abstract concepts (Dang et al., 2020; Fang, 2021). The subject of the former is mainly teachers (learners may also participate in it), that is, teachers create a learning environment including information technology to provide visual learning support for learners; The main body of the latter is learners, that is, learners abstract the concrete content into concepts and models, and express them visually. In the interpersonal field, relevant strategies include: setting up cooperative research groups, and in most schools, the research groups are carried out continuously and daily; providing internship opportunities is a way for students to strengthen and cooperate their skills in off-campus situations. Because both of them focus on the cognitive level of learning, they pay less attention to the emotional level and social and cultural attributes of learning. Especially from the perspective of “complex learning environment” it is still necessary to deeply integrate the cognitive, social and technical aspects of learning. Therefore, it is still worth further discussion to apply the above two classification theories to the evaluation of deep learning. In the research, the corresponding algorithm formulas are established for analysis, such as formulas (7)–(10) plus (11).


(7) $${\left(x+a\right)}^{n}=\overset{n}{\underset{k=0}{\sum }}a^{n-k}$$

(8) $$e^{x}=1+\frac{x}{1!}+\frac{x^{2}}{2!}+\frac{x^{3}}{3!}+\cdots$$

(9) $$\frac{\Gamma }{1}+\frac{x\cdot t^{1}}{y_{T}\dot{I}}=11\pi -Im\frac{1}{5},1$$

(10) $${∭}_{V}\left(\nabla \cdot F\right)dV={∯}_{S}F\cdot dS$$

(11) $$\begin{array}{c} \nabla \cdot \nabla \psi =\frac{\partial^{2}\psi }{\partial x^{2}}+\frac{\partial^{2}\psi }{\partial y^{2}}\\ =\frac{1}{r^{2}\sin\theta }\left[\sin\theta \frac{\partial }{\partial r}\left(r^{2}\frac{\partial \psi }{\partial r}\right)+\frac{1}{\sin\theta }\frac{\partial^{2}\psi }{\partial \varphi^{2}}\right] \end{array}$$

The deep neural network has a deep non-local learning structure, and it can learn the features in the data set with great changes from fewer samples, showing stronger feature recognition ability than the kernel method. At the same time, the learning process of RDFM method solves the over-fitting problem caused by too strong learning ability due to the introduction of regularization factors. According to Fisher’s criterion, the depth structured neural network is used to improve the discrimination of features. Deep neural network has a deep nonlocal learning structure and learns the characteristics of data sets with great changes from fewer samples. It shows stronger feature recognition ability than kernel method. At the same time, due to the introduction of regularization factor in the learning process of rdfm method, the problem of over-fitting caused by strong learning ability is solved. Experiments are carried out on various types of data sets, and the results show the necessity of using unsupervised regularization in the fine-tuning stage of deep learning. Experiments on image classification and learning low-dimensional representation of images with the depth unsupervised self-coding model realized by this nonlinear transformation method show that these transformations are helpful to learn the depth structure neural network with at least five hidden layers, which proves the effectiveness of the transformation, improves the speed of the basic random gradient learning algorithm, and helps to find a better generalized classifier. Deep neural networks such as convolution DBN and stack self-coding network have been used in speech and audio data processing, such as music artist genre classification, speaker identification, speaker gender classification and speech classification, etc., and very good learning results have been obtained (Koops and Kuebel, 2021). DBN and stack self-coding network have shown good performance in a single image recognition task, successfully used to generate a compact and meaningful image retrieval representation, and have been used in large-scale image retrieval tasks with very good results.

## Results and analysis

### Study on the influence of music appreciation on students’ mental health

The form of music appreciation teaching is mainly characterized by listening and appreciating, and the music works, because of its own structural style, are in the form of tension, which causes the conscious person to have a specific psychological reaction. Interpersonal relationship refers to the relationship between people established and developed in interpersonal communication, which reflects the contact degree of people in the depth, closeness, coordination and other psychological aspects. The vast majority of contemporary college students are only children, and they have developed the habit of self-centered thinking and dealing with problems. They show no cooperative spirit, no broad mind, and haggle over every ounce when dealing with others. Even though they are aware of the importance of interpersonal relationships, they often find it difficult to get along with others for various reasons. Music maintains mental health by promoting harmonious interpersonal relationships. Every one of us lives in the society, not an isolated existence, and personal mental health is inseparable from the society. The comprehensive functions of music appreciation are reflected in picture appreciation, field appreciation, self-appreciation and creative appreciation. Music appreciation can promote individuals’ mental health and alleviate their negative emotions. This conclusion has been confirmed by many psychological studies. In modern society, a large number of people are in a tense state of life, so physical and mental relaxation plays a great role in mental health. However, not every kind of music can make people relax. Music that can make people relax should generally meet the following requirements: the rhythm is less than the heartbeat, and the rhythm changes little. Music can include flutes, strings, guitars, etc., preferably concertos with soft melodies. With the formation of human self, there is a need to be cared for by others, that is, in one’s life, one hopes to feel warmth, care, sympathy, respect, recognition, etc. from related people. Listening to music can cultivate students’ sentiment, make them have noble spiritual realm, broader vision and mind, cultivate their image memory, creativity, imagination and observation, improve their understanding, perception, emotion and other abilities, and form a correct aesthetic view. Psychological energy is the psychological power, which can make people realize their subjectivity and needs, the courage, impulse willpower, feelings and emotions with various characteristics, etc. Psychological energy can affect the balanced and coordinated development of the dynamic system of human psychology, and it is the key. The psychological energy of college students exists in the form of tension, and the sound of music also stimulates the appreciators in the form of tension. The two kinds of tension combine and influence each other, so that students’ psychological energy can move and play smoothly. The sound of music forms a complete musical work through its constituent elements, such as loudness, pitch and timbre. Music stimulates human sensory organs through various elements of sound. In the teaching of music appreciation, teachers guide students to have emotional experience of music, and guide students to comprehend and feel through all kinds of information and details in music, so that students can integrate their emotions into music. At present, college students are full of energy, rich in knowledge and mature in physiology, and their emotional world is very colorful.

**Figure 4:**
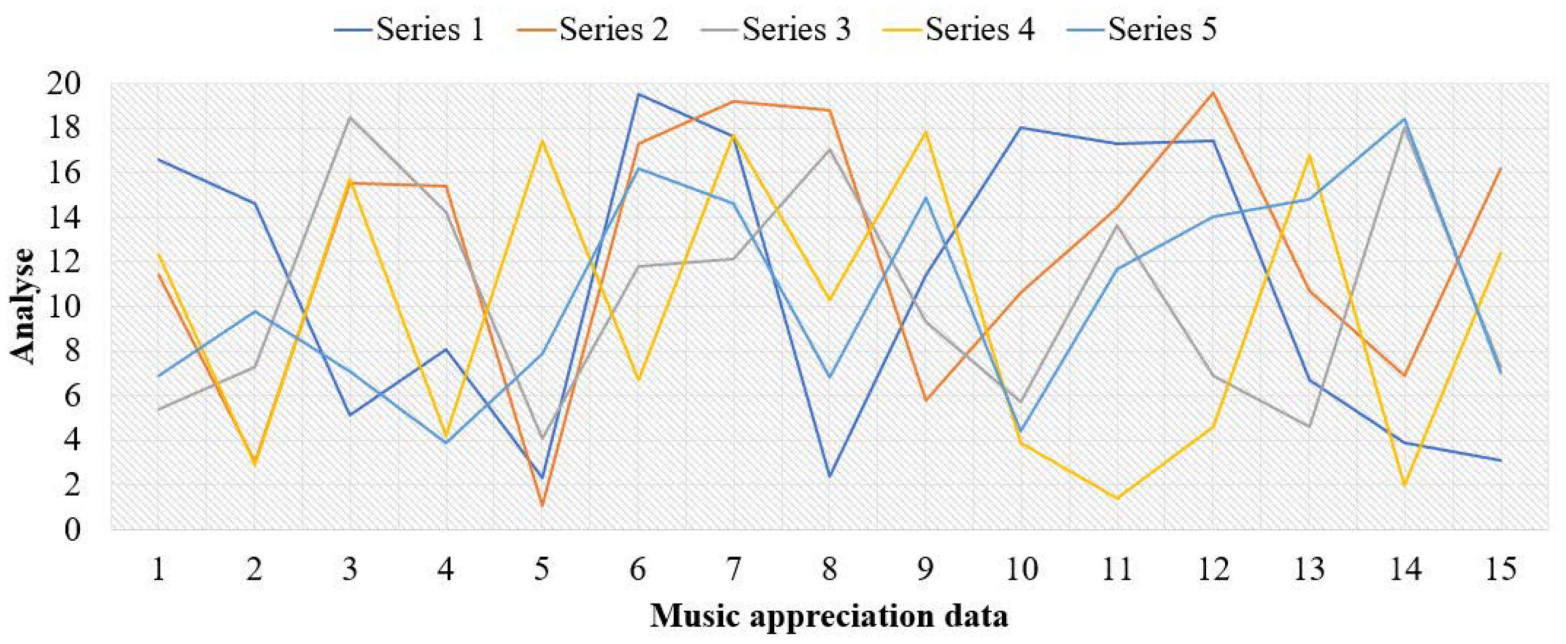
Data map of music appreciation influence.

**Figure 5:**
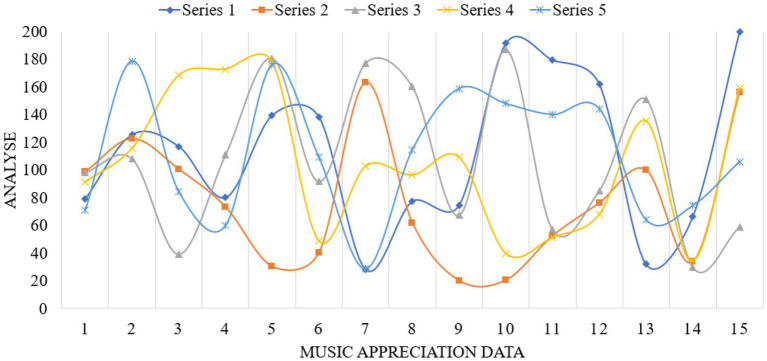
Data map of music appreciation research.

**Figure 6:**
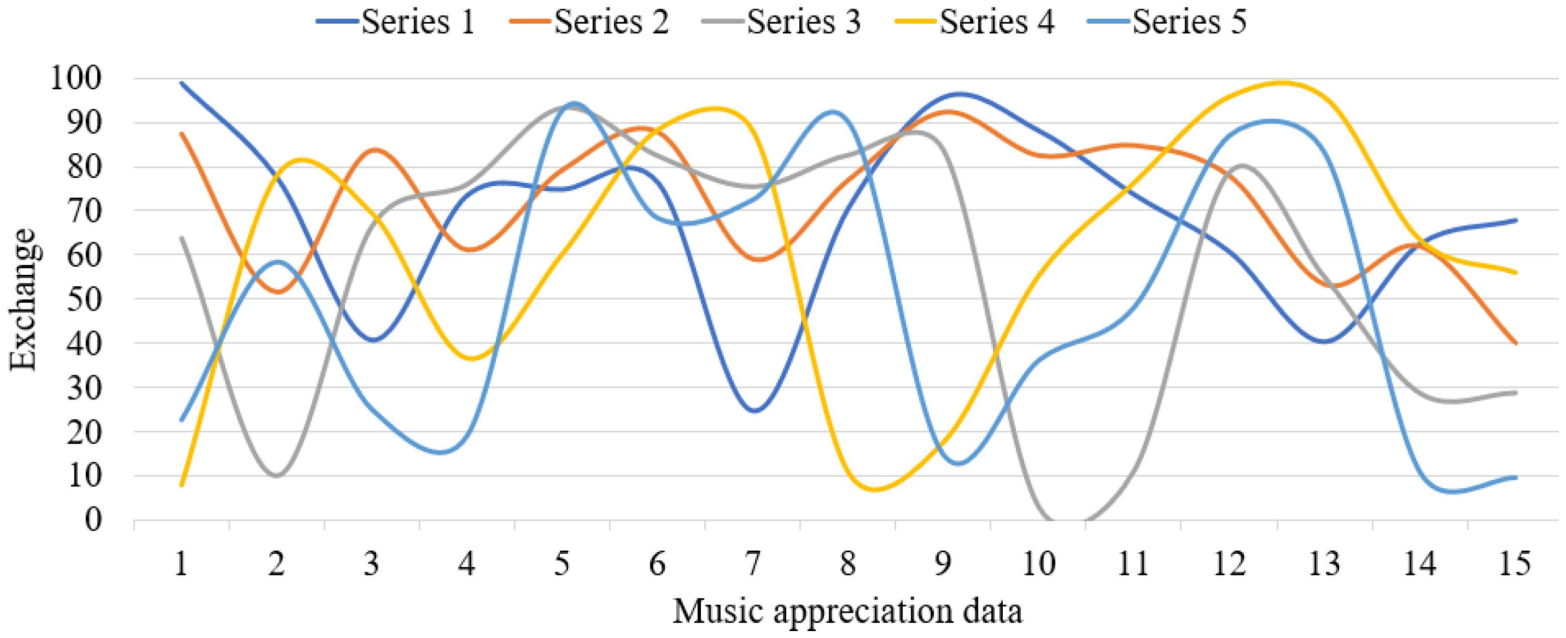
Music appreciation data map.

This paper investigates a vocational and technical college and a conservatory of music. These schools are representative, objective and universal, and they are all higher vocational colleges. The investigation lasted 4 months. Thousand questionnaires were distributed to the students of the above four schools, and 900 were recovered, with a recovery rate of 90%. Among them, there are 900 valid questionnaires, with an effective rate of 100%. In the study, the corresponding data graphs are established for analysis, as shown in Figures 4–6.

From the above data graph, it can be known that music appreciation has a great influence on psychological activities, up to about 54.3%. Personality is composed of three parts: id, ego and superego. I am the original self, which refers to the original self, including all the basic desires, vitality and impulses required for production. As the source of psychological energy, the ID only needs to be happy and avoid suffering. Happiness is the fundamental principle of the ID’s behavior, completely outside the social moral norms. The psychological energy of self is mostly consumed in the suppression and control of id. Anything that can become conscious is in the self, but it also exists in the unconscious in the self. It is the existence and awakening of self-consciousness. Self can separate desire from fantasy, endure tension and compromise, and change with time. In music appreciation, the appreciator’s self will be strengthened. Music appreciation teaching is a way to further deepen the influence of personality. On the basis of the original value of music, the harmonious development of people’s psychology can be further realized. Idealized personality must be the result of highly harmonious development of internal psychology. Pursuing truth, goodness and beauty also points out the direction for realizing the harmony of internal psychology. Superego is an ideal part of personality structure and a perfect self. I strive for perfection, and it does not care about reality or happiness. Because of the guidance of the superego, the heart will achieve a harmonious state, thus enabling people to live better in real life. Music appreciation can provide a beneficial direction for college students’ superego. Music appreciation is an important part of aesthetic education. To complete music appreciation education with high quality, the most direct thing is to have an excellent music appreciation teacher. An excellent music teacher should have all the excellent qualities of all teachers, including solid basic music skills and professional accomplishment. We should also have many other excellent qualities, such as innovative spirit, profound knowledge, correct aesthetics, etc. Teachers who have graduated from music colleges are more concentrated in their professional fields, and lack relevant knowledge of psychology. Teachers who have graduated from local comprehensive universities and normal colleges also have the same problems to varying degrees. Music psychology is based on general psychological phenomena, and constantly evolves in the process of the emergence, occurrence and development of music consciousness. Music psychology is not innate. It needs to gradually form music consciousness and psychological process with personality characteristics in the actual music activities and education, and in the process of continuous improvement of the brain. The process of continuous learning and acceptance in the growth stage is also the process of the formation of musical psychology. From infancy to adulthood and then to old age, it has experienced the whole process of occurrence, development and decline. Teachers of music majors should master and understand certain knowledge of psychology if they want to improve the mental health of higher vocational college students through music. Under the guidance of the syllabus of music education, corresponding teaching plans and requirements should be formulated for higher vocational college students of the same grade or different grades, so that students can study systematically in a planned way. In the teaching of music appreciation, we should learn from the advanced music ideas, and integrate the most advanced teaching ideas in the world into our own teaching methods, so as to enrich our teaching content and make our teaching methods more vivid. Music appreciation teachers can get the latest music ideas and know the latest research results by reading the latest professional music journals and magazines, so as to serve their own music appreciation teaching.

### The psychological impact of music education on students

**Figure 7:**
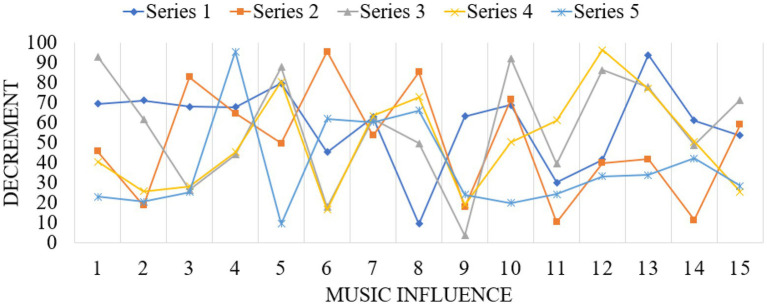
Data map of music education impact.

**Figure 8:**
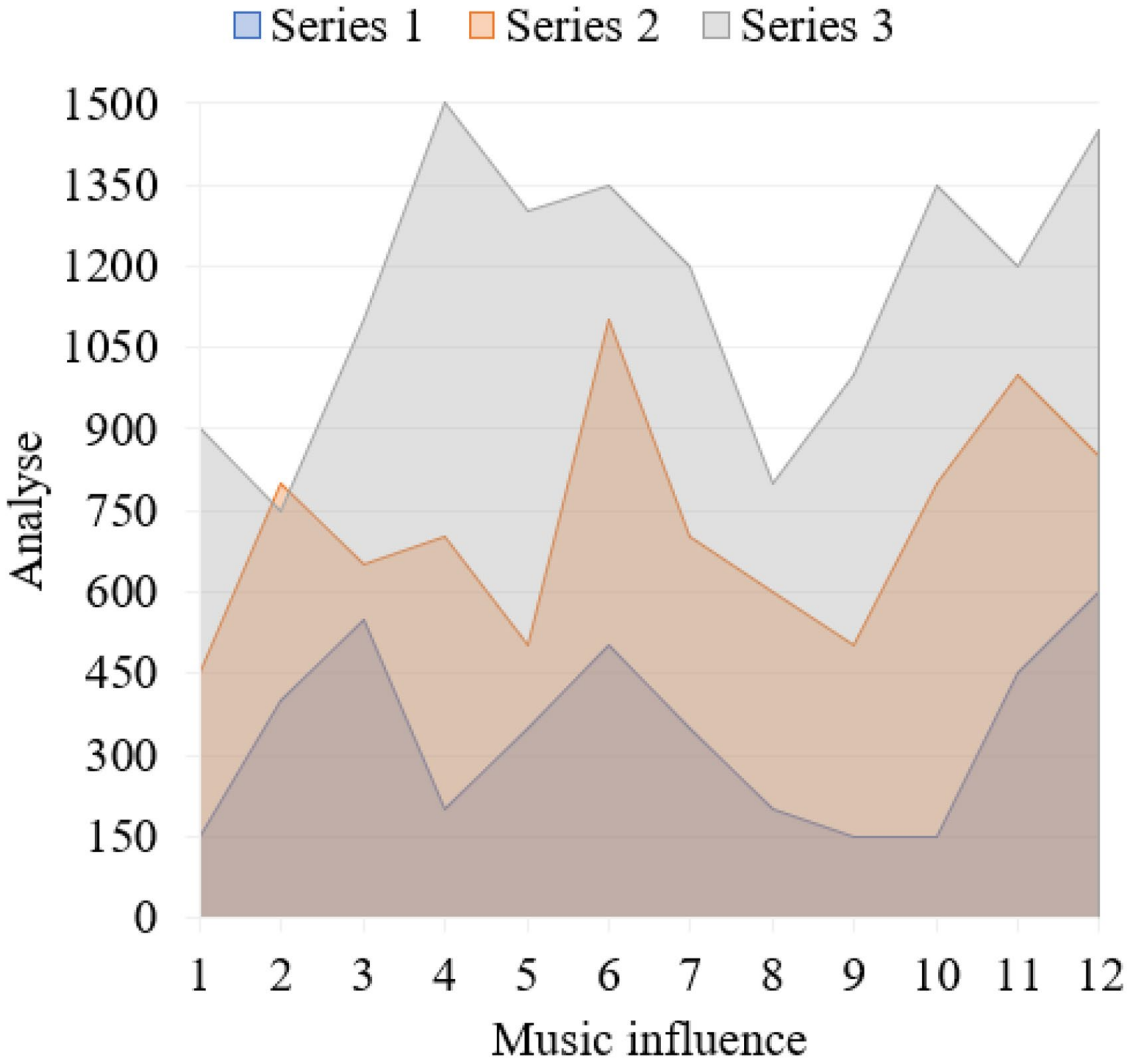
Data map of music education research and analysis.

**Figure 9:**
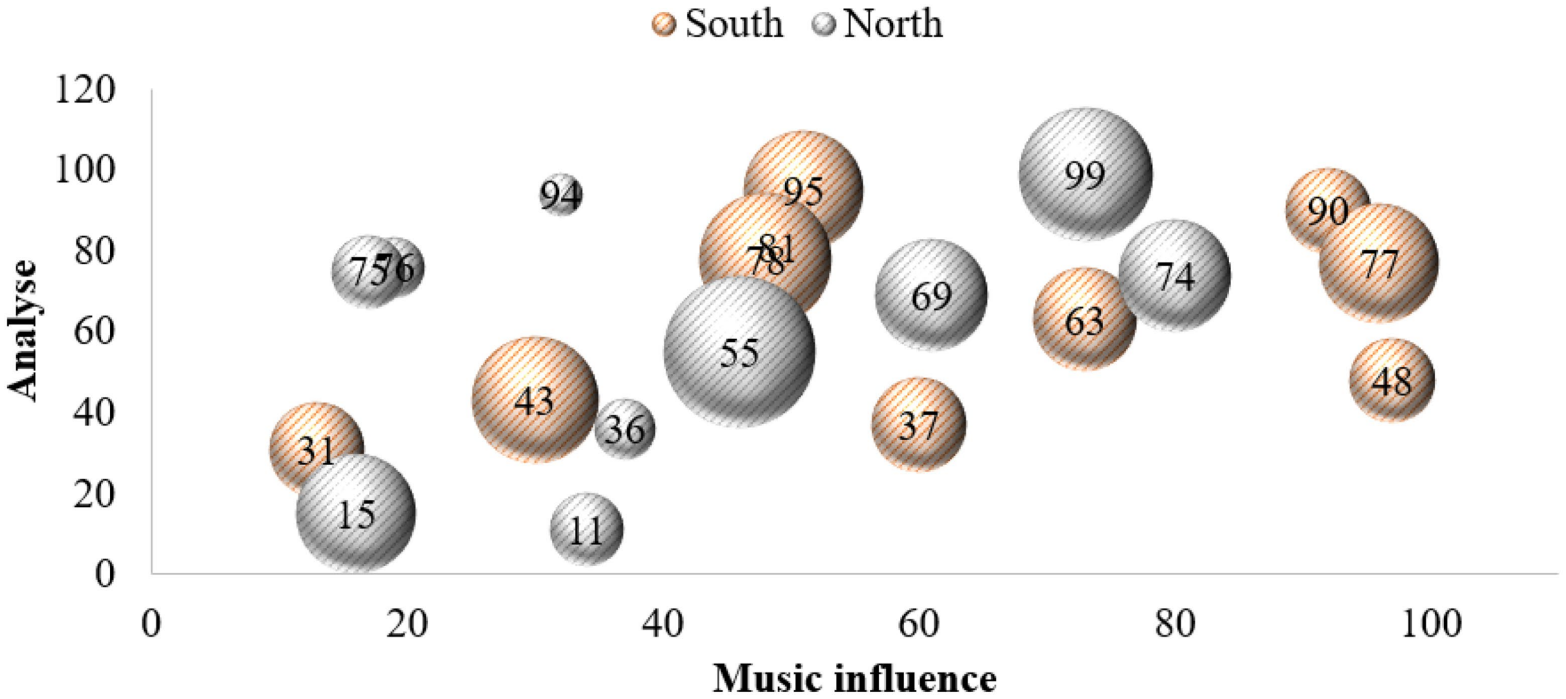
Data map of music education impact effect.

To study students’ mental health, we have to talk about psychology. Psychology is a subject with many branches. Psychology and music psychology provide theoretical basis for music education to solve students’ mental health problems from different angles. In the early stage, young people can not only look at themselves objectively, but also express themselves clearly, defend themselves sensitively, and cherish themselves, forming a rational self-consciousness. The ideal self and the realistic self are still facing the crisis of division, and self-affirmation and self-denial often conflict. Chronic anxiety symptoms develop slowly, but persist, usually for a long time. In terms of emotional disorders: I often feel distressed, self-reproached, always think of the disadvantages and exaggerate the difficulties when something happens. No moaning, often accompanied by fatigue, chest tightness, shortness of breath, irritability, sensitivity, frequent anxiety, loss of hands and feet, upset, restless, rubbing hands and feet, an unbearable discomfort, anxiety about one’s health, head swelling, face fever, etc. The fierce competition for talents in modern society often leads to college students’ involuntary study pressure and employment pressure, which gather in their hearts and cause obvious anxiety. Accordingly, college students will have the need to seek to relieve their inner pressure and anxiety. Music education is undoubtedly a “good medicine” to relieve college students’ psychological pressure. Music teaching can teach students about the height, intensity, timbre, melody, harmony, rhythm and other aspects of music, so that students can have a more comprehensive understanding of music, and then they can choose their own music independently, and enjoy their body and mind with the help of music. Excellent music plays a positive role in shaping a sound personality. With the help of classroom teaching and extracurricular music activities, music teaching provides a platform for college students to contact and appreciate excellent music works. In the research, the corresponding data graphs are established for analysis, such as Figures 7–9.

The beauty of music lies in being able to depict an exciting artistic conception through the intangible emotion of music. Compared with other arts, music is closer to nature, it is easier to express one’s feelings, and it can make the appreciator’s mind deeply moved, which can exert a subtle influence. As an auditory art, music exists through auditory feelings. Let students feel the aesthetic effect in listening, which can better promote students’ imagination beauty, and make music exert great influence on their minds through image, so that students’ body and mind are in a benign and healthy state of development. Because music originated from a long time ago in human society, it was originally for human beings to express their emotions and vent their bad emotions, so music is called “the language of emotions”. Music can penetrate into the deepest part of the soul with the strongest power. If the way of education is suitable, they will infiltrate the soul with beauty and beautify it. If there is no such proper education, the soul will be ugly. Undoubtedly, good music teaching methods can help middle school students enrich their inner feelings and perfect their personality charm. It can also enable middle school students to feel the wonder and beauty of the world. For many middle school students, the emotions conveyed by good music are like guides, leading middle school students’ thoughts to a positive perspective. Listening to good music often can make middle school students calm down and learn cultural knowledge, and can sublimate students’ emotions. Implementing music education is a pleasure in itself, and under its influence, students’ pressure from all sides will be relieved and released, thus reducing their anxiety in learning. One of the standards of mental health is to have good interpersonal relationships. People with mental health can always keep good contact with the society and others, and can correctly know and understand the society and others. Music appreciation teaching is also aesthetic education. Teachers should, according to the characteristics of the times, the age of students and other factors, on the premise of maintaining the richness of classroom teaching content, choose works with strong ideological content and typical representative significance for students to enjoy. Music psychology is a branch of psychology that studies and explains people’s music experience and music behavior from primitive (newborn) to advanced level, based on psychological theory, absorbing physiology, physics, genetics, anthropology, aesthetics and other related theories, and adopting the method of experimental psychology.

## Summary

Music is closely related to students’ mental health. Music education can promote students’ mental health, enable students to relax themselves, express their feelings, release bad emotions, build harmonious interpersonal relationships, and help students establish a good mental health system. In terms of influencing factors and strategies to promote students’ deep learning, we should apply the research results to specific teaching situations with the help of advanced digital technology, and strive to combine theory with practice. The mental health of college students is an important part of quality education in colleges and universities, and music education plays an important role in the implementation of quality education. It is diversified, multi-faceted, multi-level and repeated to influence and educate people. The effects of music appreciation on college students’ mental health are analyzed and discussed in detail in this paper: the effects on college students’ mental health, mental energy, psychological structure and so on. The idea that music promotes people’s mental health has existed for a long time, and the related researches in the fields of music psychology, music therapy and music education psychology have become quite mature systems. However, for the specific group of primary and secondary school students, it should be said that it is relatively rare to consider using music education to promote their mental health. This paper summarizes the advantages of deep learning over shallow learning, explains the necessity of introducing deep learning, describes the data representation of deep learning and several typical deep learning models, such as convolutional neural network, DBN and stack self-coding network, explains the reasons that may cause difficulties in deep learning training, introduces effective training methods, and summarizes the new progress of deep learning research in recent years from four aspects: initialization method, selection of network layers and activation functions, model structure, learning algorithm and practical application. However, this study lacks large-scale data for training, and obtains a large number of more representative characteristic information. So as to classify and predict the samples, and improve the accuracy of classification and prediction. Therefore, there are certain limitations, and further analysis is needed in the future.

## Data availability statement

The raw data supporting the conclusions of this article will be made available by the authors, without undue reservation.

## Author contributions

TW: conceptualization, methodology, formal analysis, writing—original draft. YZ: conceptualization, validation, data curation, writing—original draft. MY: methodology, validation, data curation, writing—original draft. All authors contributed to the article and approved the submitted version.

## Funding

Research and practice of general education of Aesthetic Education in Colleges and Universities from the perspective of “Internet +” (SJGZ20210071).

## Conflict of interest

The authors declare that the research was conducted in the absence of any commercial or financial relationships that could be construed as a potential conflict of interest.

## Publisher’s note

All claims expressed in this article are solely those of the authors and do not necessarily represent those of their affiliated organizations, or those of the publisher, the editors and the reviewers. Any product that may be evaluated in this article, or claim that may be made by its manufacturer, is not guaranteed or endorsed by the publisher.
